# Synthesis of High-Molecular-Weight Polyhydroxyalkanoates by Marine Photosynthetic Purple Bacteria

**DOI:** 10.1371/journal.pone.0160981

**Published:** 2016-08-11

**Authors:** Mieko Higuchi-Takeuchi, Kumiko Morisaki, Kiminori Toyooka, Keiji Numata

**Affiliations:** 1 Enzyme Research Team, Biomass Engineering Research Division, RIKEN Center for Sustainable Resource Science, Wako, Saitama, Japan; 2 Mass Spectrometry and Microscopy Unit, Technology Platform Division, RIKEN Center for Sustainable Resource Science, Yokohama, Kanagawa, Japan; Tsinghua University, CHINA

## Abstract

Polyhydroxyalkanoate (PHA) is a biopolyester/bioplastic that is produced by a variety of microorganisms to store carbon and increase reducing redox potential. Photosynthetic bacteria convert carbon dioxide into organic compounds using light energy and are known to accumulate PHA. We analyzed PHAs synthesized by 3 purple sulfur bacteria and 9 purple non-sulfur bacteria strains. These 12 purple bacteria were cultured in nitrogen-limited medium containing acetate and/or sodium bicarbonate as carbon sources. PHA production in the purple sulfur bacteria was induced by nitrogen-limited conditions. Purple non-sulfur bacteria accumulated PHA even under normal growth conditions, and PHA production in 3 strains was enhanced by nitrogen-limited conditions. Gel permeation chromatography analysis revealed that 5 photosynthetic purple bacteria synthesized high-molecular-weight PHAs, which are useful for industrial applications. Quantitative reverse transcription polymerase chain reaction analysis revealed that mRNA levels of *phaC* and *PhaZ* genes were low under nitrogen-limited conditions, resulting in production of high-molecular-weight PHAs. We conclude that all 12 tested strains are able to synthesize PHA to some degree, and we identify 5 photosynthetic purple bacteria that accumulate high-molecular-weight PHA molecules. Furthermore, the photosynthetic purple bacteria synthesized PHA when they were cultured in seawater supplemented with acetate. The photosynthetic purple bacteria strains characterized in this study should be useful as host microorganisms for large-scale PHA production utilizing abundant marine resources and carbon dioxide.

## Introduction

Polyhydroxyalkanoate (PHA) is a biopolyester that functions both as an intracellular carbon and energy storage molecule as well as a sink for reducing redox potential [1, 2]. PHA has garnered attention as an alternative to petroleum-derived plastics due to its biodegradability and biocompatibility [3]. One of the best studied types of bacteria in the context of PHA production is *Ralstonia eutropha* H16, and recombinant strains of this bacterium are used in numerous industrial bioprocesses [4, 5]. Although efforts have been made to reduce the price of PHA, the cost of the necessary carbon sources, such as sugars or plant oils, is still high compared with petroleum-derived plastics. To solve this problem, some researchers have focused on direct fixation of CO_2_ to PHAs via photosynthesis in an attempt to reduce the price of PHA production. For example, transgenic higher plants have been modified to produce higher levels of PHA [6]. Furthermore, several strains of cyanobacteria have been reported to contain active PHA synthases and to accumulate 3-hydroxybutyrate (3HB) [7, 8]. However, high PHA productivity in higher plants or cyanobacteria has yet to be achieved.

In addition to higher plants and cyanobacteria, anoxygenic photosynthetic bacteria have also been reported to accumulate PHA [9]. Anoxygenic photosynthetic bacteria can be divided into the following five categories based on pigments: electron donors, and aerobic/anaerobic condition: purple sulfur bacteria, purple non-sulfur bacteria, green sulfur bacteria, green non-sulfur (filamentous) bacteria and aerobic photosynthetic bacteria. Unlike higher plants and cyanobacteria, anoxygenic photosynthetic bacteria extract electrons from molecules other than water, such as organic compounds, sulfur compounds and hydrogen. The majority of anoxygenic photosynthetic bacteria can grow as photoautotrophs or photoheterotrophs in the light, and some members can grow in the dark as chemoheterotrophs. These bacteria can utilize various types of organic compounds as carbon sources. Due to these characteristics, photosynthetic bacteria have been tested for use in a variety of applications, including purification of industrial wastewater and hydrogen production [9].

PHA production has been studied in a small number of freshwater purple non-sulfur bacteria strains such as *Rhodospirillum rubrum* [10], *Rhodobacter sphaeroides* [11] and *Rhodopseudomonas palustris* [12], with a focus on carbon source, culture conditions and PHA yield. *Rhodospirillum rubrum* is the best characterized strain with respect to PHA production, and it showed 50 wt% PHA content by dry cell weight when butyrate was used as the sole carbon source [10]. It was reported that *Rhodobacter sphaeroides* and *Rhodopseudomonas palustris* accumulated PHA levels of 60–70 wt% [11] and 4 wt% [12], respectively. In contrast, studies of PHA production in marine photosynthetic bacteria are limited to only a few strains [13, 14]. The marine purple non-sulfur bacterium *Rhodovulum sulfidophilum* reportedly possesses the ability to synthesize poly[(*R*)-3-hydroxybutyrate] (PHB) up to 50 wt% of its dry weight [13].

Marine bacteria are attracting attention as new biotechnological resources, and it is expected that they will yield a wealth of new bioactive compounds [15–17]. However, the potential of these bacteria remains largely unexplored. The utilization of marine bacteria has a number of potential advantages in terms of commercial applications. For example, when marine bacteria are used for commercial scale cultivation, sterilized seawater can be used as a culture medium without the need for a synthetic medium, leading to considerable savings on fresh water. Additionally, bacterial contamination is a serious problem for commercial production involving microbial fermentation. However, the high salt concentration of seawater inhibits contamination from the air during the cultivation of marine bacteria. Considering those potential advantages, we focused on marine photosynthetic purple bacteria as a host microorganism for PHA production. In this study, we evaluated PHA production in marine photosynthetic purple bacteria and characterized the resultant PHAs, and we found them to be suitable new host microorganisms for photosynthetic PHA production.

## Results and Discussion

We cultured 16 purple sulfur bacteria and 17 purple non-sulfur bacteria strains, as shown in S1 Table, yielding a total of 33 strains of photosynthetic purple bacteria that were tested for growth in liquid culture. Of these strains, 3 purple sulfur bacteria and 9 purple non-sulfur bacteria showed relatively high growth in liquid culture (Table 1). These 12 strains were further characterized as candidates for PHA production. One candidate strain, *Rdv*. *sulfidophilum*, was previously reported to accumulate PHA [14], whereas the remaining 11 strains had not been previously characterized for PHA production. We searched the public database and found that whole genome sequences have been determined only in *Rdv*. *sulfidophilum*. We found the *phaC* gene encoding PHA synthase which belongs to the class I in the genome of *Rdv*. *sulfidophilum*.

**Table 1:** Candidate strains of photosynthetic purple bacteria.

Sulfur type	Resource No.	Organism	Original marine area
Sulfur	DSM5653	*Thiohalocapsa marina*	Mediterranean Sea
Sulfur	JCM14889	*Thiophaeococcus mangrovi*	Orissa, India
Sulfur	JCM13911	*Marichromatium bheemlicum*	Bhimunipatnam, India
Non-sulfur	DSM2698	*Afifella marina*	Kagoshima, Japan
Non-sulfur	DSM4868	*Rhodovulum euryhalinum*	Russia
Non-sulfur	JCM13589	*Rhodovulum imhoffii*	Bhimunipatnam, India
Non-sulfur	ATCC35886	*Rhodovulum sulfidophilum*	Groningen, Netherlands
Non-sulfur	ATCC BAA1573	*Rhodovulum tesquicola*	Soda Lake, Russia
Non-sulfur	JCM13531	*Rhodovulum visakhapatnamense*	Visakhapatnam, India
Non-sulfur	ATCC BAA447	*Roseospira marina*	Arcachon Bay, France
Non-sulfur	JCM14191	*Roseospira goensis*	Goa, India
Non-sulfur	ATCC BAA1365	*Roseospira visakhapatnamensis*	Kakinada, India

PHA accumulation is known to be enhanced when excess carbon is present and other nutrient(s), such as nitrogen, phosphorus or sulfur, are limited [18]. In the case of photosynthetic bacteria, PHA production can be induced under nitrogen- or vitamin-limited conditions [10], [13]. In addition, acetic acid was shown to be an efficient carbon source for PHA production in photosynthetic bacteria [13, 19]. Therefore, in this study, we used a nitrogen-limited medium (ammonium chloride-free) containing sodium acetate to test PHA production. Carbon dioxide was supplied by 0.1% NaHCO_3_. Twelve photosynthetic purple bacteria strains were cultured in growth medium until the cell cultures reached an approximate OD_660_ value of 1.0, indicating they were in log phase, and these cells were harvested and used as growth conditions without supplementation and depletion. Intracellular PHAs under growth condition were analyzed using GC-MS. The log phase cell cultures were diluted to an OD_660_ of 0.1 in nitrogen-limited media supplemented with 0.5% sodium acetate and/or 0.1% NaHCO_3_. After 7 days, just prior to stationary phase, the cells were harvested, and intracellular PHA levels were characterized.

**Fig 1:**
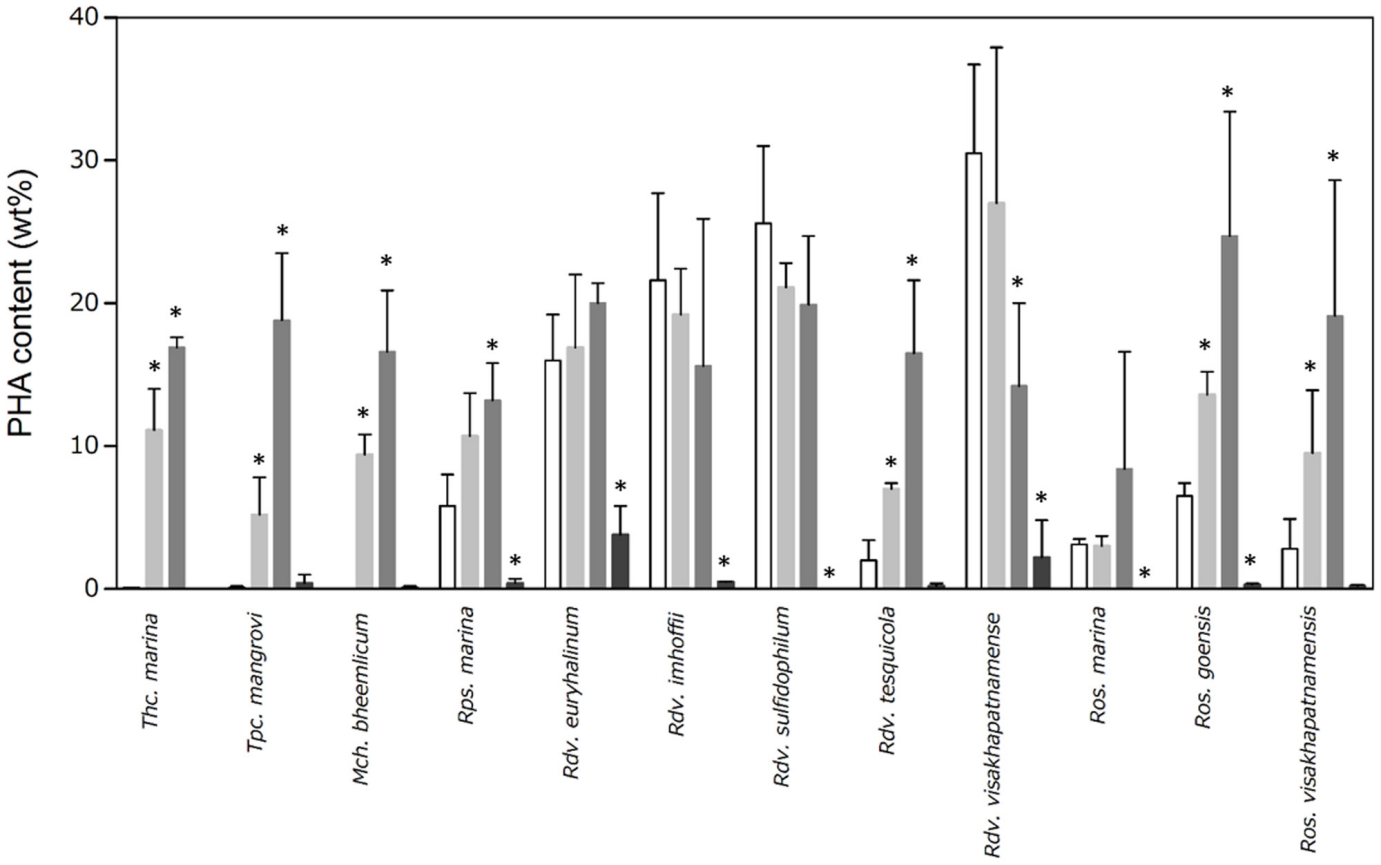
PHA content (wt%) of photosynthetic purple bacteria. Photosynthetic purple bacteria strains were cultured in growth medium until the cell cultures reached an approximate OD_660_ value of 1.0 and then diluted to an OD_660_ of 0.1 in nitrogen-limited media supplemented with 0.5% sodium acetate and/or 0.1% NaHCO_3_. After 7 days, the cells were harvested, and intracellular PHA levels were characterized using GC-MS. Intracellular PHAs under growth conditions were analyzed using cells showing approximately OD_660_ = 1.0 without supplementation and depletion. Data are the mean ± SD of at least three cultures. *Values that show significant differences compared to the growth conditions (p<0.05).

Three of the purple sulfur bacteria (*Thc*. *marina*, *Tpc*. *mangrove*, and *Mch*. *bheemlicum*) accumulated less than 0.1 wt% PHA by dry cell weight under growth conditions (Fig 1, white bars). In contrast, 9 purple non-sulfur bacteria strains accumulated PHA levels ranging from 2.0 to 30 wt%, even under growth conditions. Intracellular PHAs were analyzed when the bacteria were grown in nitrogen-limited media containing both sodium acetate and NaHCO_3_. Three purple sulfur bacteria strains accumulated more PHAs (5 to 11 wt%) under these conditions compared with growth conditions (Fig 1, light gray bars). Although increased PHA production was observed in 3 purple non-sulfur bacteria (*Rdv*. *tesquicola*, *Ros*. *goensis*, and *Ros*. *visakhapatnamensis*), the degree of PHA induction was not remarkable compared with the purple sulfur bacteria. PHA accumulation was not enhanced in the remaining 6 purple non-sulfur bacteria (*Rps*. *marina*, *Rdv*. *euryhalinum*, *Rdv*. *imhoffii*, *Rdv*. *sulfidophilum*, *Rdv*. *visakhapatnamense*, and *Ros*. *marina*).

In some bacterial species, it has been proposed that nitrogen fixation competes with PHA formation for reducing equivalents [4]. Photosynthetic bacteria have the ability to fix nitrogen due to the presence of nitrogenase enzymes. We identified two nitrogenases in the genome of *Rdv*. *sulfidophilum* [20, 21], suggesting that it has nitrogen fixation activity. Some strains showed higher CDW under nitrogen-limited conditions compared to growth condition (S1 Fig), implying that photosynthetic bacteria have nitrogen fixation activity, even though direct assay related to nitrogen fixation such as nitrogenase catalyzed acetylene reduction experiment is needed to clear the nitrogen fixation activity. Based on these observations, we suggest that PHA production was not enhanced under nitrogen-limited conditions due to strong nitrogen fixation activity in the 6 purple non-sulfur bacteria. Other deficiency conditions, such as phosphorus-, sulfate- and trace element-limited conditions, have been used to induce PHA production in other bacteria [4, 18, 22]. Indeed, PHA production was shown to be induced in *Rdv*. *sulfidophilum* when grown in vitamin-free medium [13]. Therefore, the optimization of PHA induction conditions is necessary to maximize PHA productivity in photosynthetic bacteria in the future.

PHA production was also investigated under nitrogen-limited culture conditions with only sodium acetate or NaHCO_3_ as a carbon source (Fig 1, acetate; dark gray bars, NaHCO_3_; black bars). Eleven photosynthetic purple bacteria showed PHA accumulation, which ranged from 8 to 25 wt% under nitrogen-limited culture conditions containing acetate as the carbon source. In one strain, *Rdv*. *visakhapatnamense*, PHA content was significantly decreased. PHA increased significantly under nitrogen limitation by the addition of only acetate in *Rps*. *marina* although there was no significant increase under nitrogen limitation with both 0.5% acetate and 0.1% NaHCO_3_. We next compared PHA levels under these conditions to those from mixed carbon sources (acetate/NaHCO_3_). When acetate was used as the sole carbon source, compared with mixed carbon sources, PHA content was higher in six bacteria strains (*Thc*. *marina*, *Tpc*. *mangrove*, *Mch*. *bheemlicum*, *Rdv*. *tesquicola*, *Ros*. *goensis*, and *Ros*. *visakhapatnamensis*). PHAs are highly reduced compounds, and therefore, their accumulation affects the redox state of the cell. Indeed, it has been proposed that PHAs are important for maintaining the proper redox state in nitrogen-fixing bacteria [4]. Under mixed carbon conditions, CO_2_ could modify the intracellular redox state through photosynthetic electron transport, resulting in decreased PHA levels in these 6 photosynthetic bacteria. The PHA contents of these six bacteria significantly increased under nitrogen-limited conditions, implying that they have little or no nitrogen fixation activity. In the case of such bacteria, reducing the redox state could be used to reduce CO_2_. None of the photosynthetic purple bacteria strains could accumulate PHA (<0.1 wt% to 4 wt%) in the nitrogen-limited medium containing only NaHCO_3_ as a carbon source (Fig 1), indicating that conversion of NaHCO_3_ into PHA by photosynthetic purple bacteria was quite low under this condition. As a result, the redox state was not optimal, and the concentration of NaHCO_3_ was not sufficient for PHA production.

The cell dry weights (CDW) of the 12 strains were measured under all conditions (S1 Fig). CDW was low (less than 500 mg L^-1^ culture) when the majority of the photosynthetic bacteria were grown in nitrogen-limited medium containing only NaHCO_3_. PHA content is expressed as the percentage of CDW per 1 L of cell culture (S2 Fig). Among all strains under all conditions, *Rdv*. *visakhapatnamense* showed the highest PHA content (302 ± 42 mg/L culture) under growth conditions. Five photosynthetic purple bacteria (*Mch*. *Bheemlicum*, *Rdv*. *euryhalinum*, *Rdv*. *imhoffi*, *Rdv*. *sulfidophilum*, and *Rdv*. *visakhapatnamense*) showed yields higher than 200 mg/L in PHA culture. Brandl *et al*. reported production levels of 500 mg/L PHA in *R*. *sphaeroides* and 390 mg/L PHA in *Rhodospirillum rubrum* [10]. Although further optimization of growth conditions will be required to increase PHA yield, several of the tested photosynthetic purple bacteria are promising host strains for PHA production.

**Fig 2:**
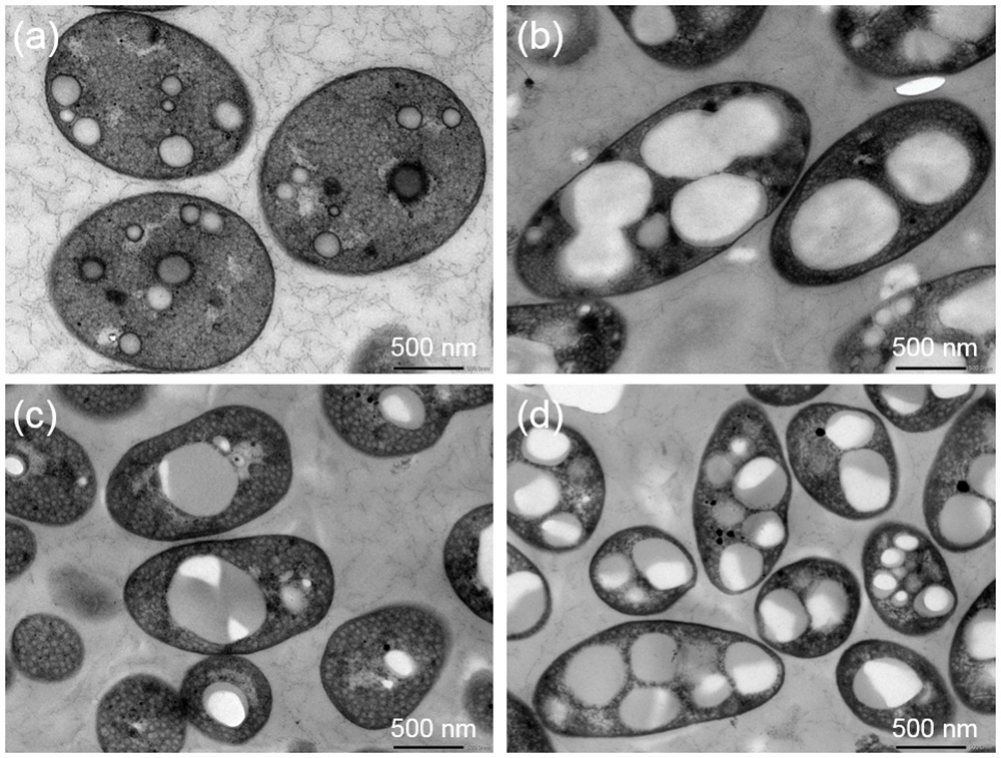
TEM images of intracellular PHA granules in *Mch*. *bheemlicum* and *Rdv*. *visakhapatnamense*. *Mch*. *bheemlicum* cells were cultured in growth medium (a) and in nitrogen-limited medium containing sodium acetate (b). *Rdv*. *visakhapatnamense* cells were cultured in growth medium (c) and in nitrogen-limited medium containing sodium acetate (d).

PHAs are found in the cytoplasm as insoluble inclusions called PHA granules. *Mch*. *bheemlicum* (purple sulfur bacteria) and *Rdv*. *visakhapatnamense* (purple non-sulfur bacteria) were cultured in growth media and then transferred to nitrogen-limited media containing sodium acetate. The PHA granules of these photosynthetic purple bacteria were then investigated by transmission electron microscopy (TEM) (Fig 2). In the cells of *Mch*. *bheemlicum*, no granules were observed under growth conditions (Fig 2a). In contrast, most cells harbored multiple large PHA granules under nitrogen-limited conditions (Fig 2b). These observations are consistent with low PHA content under growth conditions and increased PHA content under nitrogen-limited conditions (Fig 1). The diameters of the PHA granules were approximately 0.59 ± 0.14 μm, which is comparable to the average cell diameter of most bacterial species (0.4 to 1 μm). In the majority of *Rdv*. *visakhapatnamense* cells, only a single large PHA granule was observed under growth conditions (Fig 2c). In contrast, most cells contained numerous smaller PHA granules under nitrogen-limited conditions (Fig 2d). The diameters of the PHB granules were 0.52 ± 0.13 μm under growth conditions and 0.38 ± 0.07 μm under nitrogen-limited conditions. The number of PHA granules observed in *Mch*. *bheemlicum* cells was 0.06 ± 0.24 (n = 18) under growth conditions and 3.25 ± 1.28 (n = 12) under nitrogen-limited conditions. The number of PHA granules in *Rdv*. *visakhapatnamense* was 0.79 ± 0.70 (n = 14) under growth conditions and 3.50 ± 0.94 (n = 14) under nitrogen-limited conditions.

**Fig 3:**
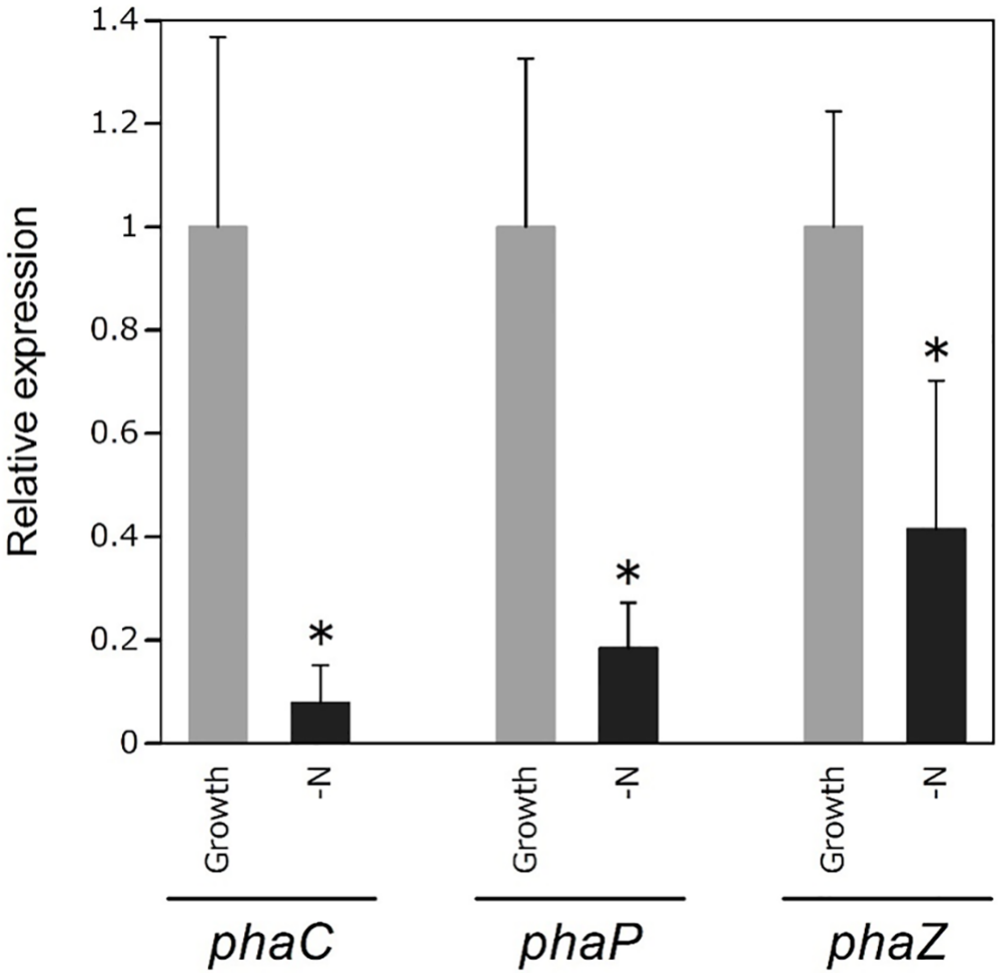
Expression levels of *phaC*, *phaP* and *phaZ* genes under nitrogen-limited conditions. RNA of *Rdv*. *sulfidophilum* was extracted under growth condition which cells were in the log phase and nitrogen-limited conditions which cells were cultured in nitrogen-limited medium for 7 days. *rpoD* gene was used as a housekeeping gene for normalizing the expression of target genes. The expression levels were shown as the relative values compared with those of growth conditions. *Values with significant differences compared with the growth conditions (*p*<0.05). Data are the mean ± SD from at least three cultures.

PHA granules are known to be coated by various granule-associated proteins [23, 24] In particular, the proteins that coat PHA granules are PHA synthase (PhaC), PHA depolymerase (PhaZ), repressor protein (PhaR) and PHA phasin (PhaP) [25–27]. The expression level of PhaP is known to affect the size and number of PHA-granules [28, 29]. *R*. *eutropha* expresses several copies of the *phaP* gene and contains large numbers of small PHA granules [29, 30]. The mRNA levels of *phaP* gene was analyzed by quantitative reverse transcription polymerase chain reaction (RT-PCR) under nitrogen-limited and acetate supplemented conditions (Fig 3). *Rdv*. *sulfidophilum* cells were used for expression analysis because whole genome sequences have been determined only in this strain. The mRNA levels of *phaP* gene was decreased under nitrogen limited conditions contrary to expectation. Changes in the number and size of PHA granules might be regulated by factors other than the levels of PhaP in photosynthetic purple bacteria. Further investigations will be required to clarify the mechanisms underlying the regulation of the number and size of PHA granules.

**Fig 4:**
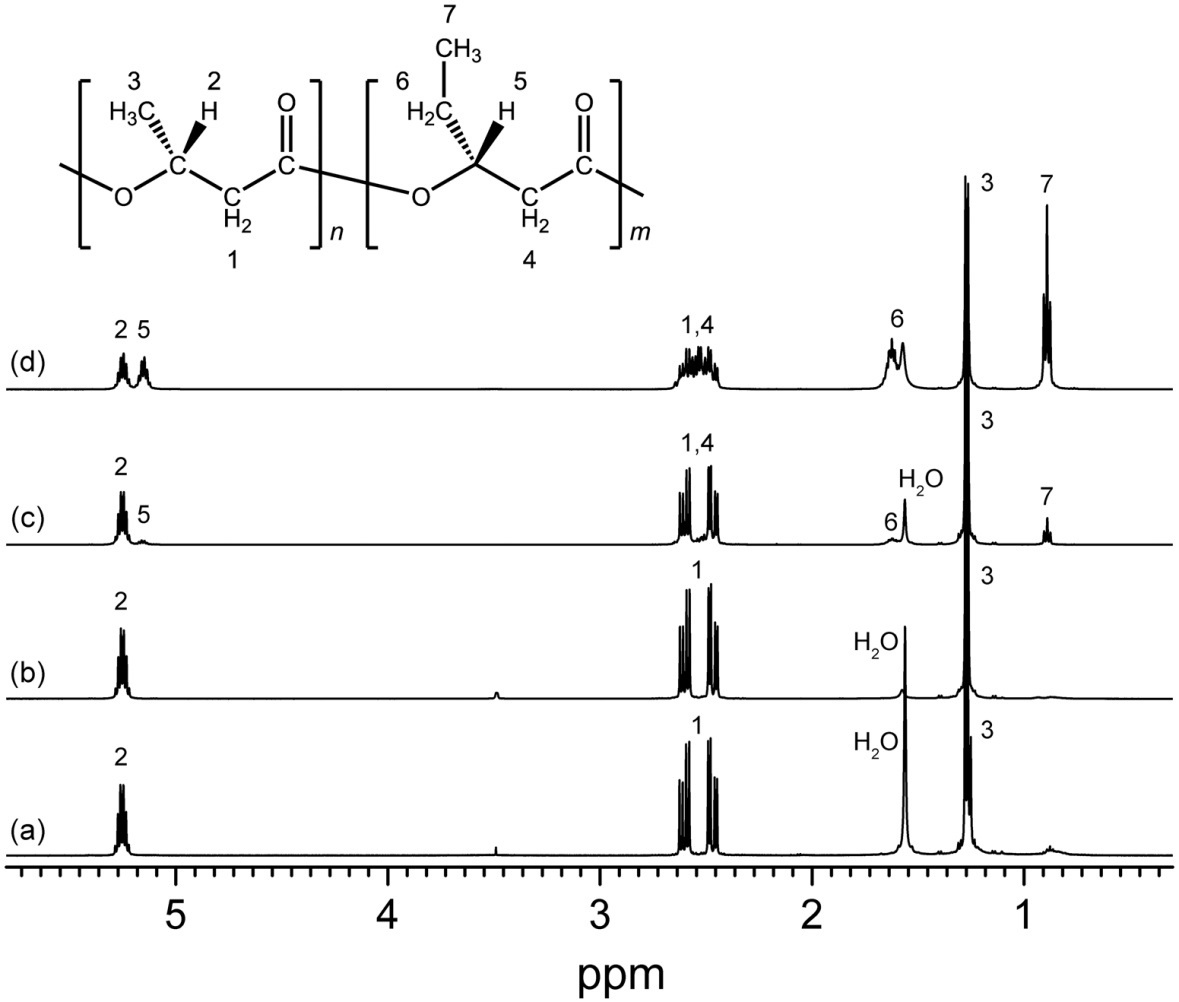
^1^H-NMR spectra of purified PHA. ^1^H-NMR analysis was carried out using purified PHA from the cells of *Thc*. *marinavi* (a) *Mch*. *bheemlicum* (b), *Rdv*. *tesquicola* (c) and *Ros*. *goensis* (d).

The chemical structures of the synthesized PHAs were determined using ^1^H NMR on chloroform-extracted samples from freeze-dried cells (Fig 4). The ^1^H NMR spectra showed the characteristic peaks for 3HB and 3HV. GC-MS analysis further revealed that purple sulfur bacteria (*Thc*. *marina*, *Tpc*. *mangrove*, and *Mch*. *Bheemlicum*) synthesized the homopolyester of 3HB under all conditions (Table 2). In contrast, all of the purple non-sulfur bacteria were able to produce copolyesters containing 3HB and 3HV units. This has also been observed in purple sulfur bacteria isolated from freshwater habitats [19]. In purple non-sulfur bacteria, the PHA compositions were variable depending on strain and carbon source. Five purple non-sulfur bacteria (*Rps*. *marina*, *Rdv*. *euryhalinum*, *Rdv*. *imhoffii*, *Rdv*. *sulfidophilum*, and *Rdv*. *visakhapatnamense*) synthesized more than 90 mol% PHB. The remaining four purple non-sulfur bacteria (*Rdv*. *tesquicola*, *Ros*. *marina*, *Ros*. *goensis*, and *Ros*. *visakhapatnamensis*) produced copolymers containing 25–90 mol% 3HV units, and the 3HV composition was higher under growth conditions. Thermal properties of the synthesized PHAs were analyzed by DSC (S2 Table). The *T*_g_ values were in the range of -4 to -1°C. Values of *T*_m_ were ranging from 74 to 174°C. *T*_m_ of PHBs was reported to be in the range of 160 to 175 [31]. Three purple non-sulfur bacteria (*Rps*. *Marina*, *Ros*. *marina*, and *Ros*. *goensis*) relatively lower *T*_m_, which is because these strains synthesized low-molecular-weight PHAs as described hereinafter with high 3HV composition (Table 2).

**Table 2:** PHA composition (mol%) under growth and nitrogen-limited conditions.

Organism	Growth condition		Nitrogen-limited conditions			
			0.5% acetate, 0.1% NaHCO_3_		0.5% acetate	
	3HB	3HV	3HB	3HV	3HB	3HV
*Thc*. *marina*	100	0	100	0	100	0
*Tpc*. *mangrovi*	100	0	100	0	100	0
*Mch*. *bheemlicum*	100	0	100	0	100	0
*Rps*. *marina*	96.0 ± 2.8	4.0 ± 2.8	95.9 ± 3.5	4.1 ± 3.5	93.9 ± 5.3	6.1 ± 5.3
*Rdv*. *euryhalinum*	99.0 ± 0.8	1.0 ± 0.8	99.2 ± 0.7	0.8 ± 0.7	97.2 ± 0.9	2.8 ± 0.9
*Rdv*. *imhoffii*	100	0	100	0	96.8 ± 3.2	3.2 ± 3.2
*Rdv*. *sulfidophilum*	100	0	94.9 ± 3.0	5.1 ± 3.0	92.6 ± 1.6	7.4 ± 1.6
*Rdv*. *tesquicola*	14.9 ± 2.2	85.1 ± 2.2	63.3 ± 11.1	36.7 ± 11.1	74.7 ± 11.3	25.3 ± 11.3
*Rdv*. *visakhapatnamense*	99.4 ± 1.0	0.6 ± 1.0	97.9 ± 0.9	2.1 ± 0.9	98.4 ± 1.5	1.6 ± 1.5
*Ros*. *marina*	9.8 ± 2.3	90.2 ± 2.3	49.9 ± 7.0	50.1 ± 7.0	70.6 ± 7.0	29.4 ± 7.0
*Ros*. *goensis*	15.9 ± 7.0	84.1 ± 7.0	56.2 ± 4.2	43.8 ± 4.2	34.0 ± 8.9	66.0 ± 8.9
*Ros*. *visakhapatnamensis*	20.6 ± 16.1	79.4 ± 16.1	72.1 ± 10.1	27.9 ± 10.1	69.9 ± 11.1	30.1 ± 11.1

Photosynthetic purple bacteria strains were cultured in growth medium until the cell cultures reached an approximate OD_660_ value of 1.0 and then diluted to an OD_660_ of 0.1 in nitrogen-limited media supplemented with 0.5% sodium acetate and/or 0.1% NaHCO_3_. After 7 days, the cells were harvested, and intracellular PHA levels were characterized using GC-MS. Intracellular PHAs under growth conditions were analyzed using cells showing approximately OD_660_ = 1.0 without supplementation and depletion. Data are the mean ± SD of at least three cultures.

The number-average molecular weight and PDI were determined from the GPC analysis of the PHAs (Table 3). The number-average molecular weight of the purified PHAs was between 3,000 and 994,000 g/mol, and the PDI was between 1.5 and 6.7. The PDI values were lower in photosynthetic purple bacteria that synthesized lower molecular weights of PHAs such as *Rps*. *marina*, *Ros*. *marina* and *Ros*. *goensis*. The PDI values were higher in higher molecular weight PHAs. In this study, PHAs were extracted from the cells grown in nitrogen-limited medium for 7 days. This long-term cultivation might induce the degradation of the PHA, resulting in the broad molecular-weight distributions. To evaluate relationship between the PHA content and molecular weight, we calculated the coefficient of correlation. The value was -0.05, indicating that there was no correlation between the PHA content and molecular weight. As shown in the GPC chromatogram, the molecular weights of PHA synthesized by several types of photosynthetic purple bacteria were ranged from approximately 30,000 g/mol to over 3,000,000 g/mol, which was larger than the column limit and the molecular weight standards (S3 Fig). The number-average molecular weight was greater than 500,000 g/mol in 5 photosynthetic purple bacteria (*Thc*. *marina*, *Mch*. *bheemlicum*, *Rdv*. *imhoffii*, *Rdv*. *tesquicola*, and *Rdv*. *visakhapatnamense*). In cyanobacteria, the molecular weight and PDI were reported to be 135,000 g/mol and 1.4, respectively [32]. Therefore, when comparing cyanobacteria and photosynthetic purple bacteria, some photosynthetic purple bacteria can produce PHA molecules with extremely high molecular weights.

**Table 3:** Number-average molecular weight and PDI of the purified PHAs.

Organism	Number-average molecular weight (g/mol)	PDI
*Thc*. *marina*	645 × 10^3^	3.7
*Tpc*. *mangrovi*	72 × 10^3^	2.9
*Mch*. *bheemlicum*	994 × 10^3^	5.8
*Rps*. *marina*	4 × 10^3^	1.5
*Rdv*. *euryhalinum*	447 × 10^3^	3.4
*Rdv*. *imhoffii*	570 × 10^3^	6.7
*Rdv*. *sulfidophilum*	300 × 10^3^	3.9
*Rdv*. *tesquicola*	504 × 10^3^	3.4
*Rdv*. *visakhapatnamense*	713 × 10^3^	3.4
*Ros*. *marina*	3 × 10^3^	1.7
*Ros*. *goensis*	17 × 10^3^	2.0
*Ros*. *visakhapatnamensis*	147 × 10^3^	5.8

PHA can be degraded within the cells by PhaZ, and indeed, bacterial cells lacking PhaZ, such as *E*. *coli*, can synthesize ultra-high-molecular-weight PHA molecules [33, 34]. Expression level of *phaZ* gene was slightly decreased under nitrogen-limited conditions in *Rdv*. *sulfidophilum* (Fig 3), which synthesized high-molecular-weight PHAs (Table 3). This result suggests one possibility that decreased level of *phaZ* gene induced production of high-molecular-weight PHAs. The expression levels of *phaZ* were reported to increase under nitrogen-limited conditions in *Ralstonia eutropha* [35]. PhaZ expression might not be induced by lack of nitrogen in case of *Rdv*. *sulfidophilum* due to its nitrogen fixation activity, as described above. Another possible explanation for production of high molecular weight PHAs is that the frequency of the chain transfer (CT) reaction was altered under nitrogen-limited conditions. PhaC catalyzes the CT reaction in which the PHA polymer chain is transferred from PhaC to a CT reagent, such as water or alcohol, following the polymerization of PHA [36]. Thus, the frequency of the CT reaction determines the molecular weight of PHA. The frequency of the CT reaction catalyzed by PhaC can range widely, leading to very different molecular weights for PHAs among photosynthetic bacteria. The molecular weight of PHA can be controlled by modulating the amount of PhaC in *E*. *coli*, and lower expression of PhaC leads to PHAs of higher molecular weight [37]. In this study, expression level of *phaC* gene was lower under nitrogen-limited conditions in *Rdv*. *sulfidophilum* (Fig 3), leading to the formation of high-molecular-weight PHAs due to lower CT reaction frequency.

Molecular weight is one of the most important properties of polymeric materials, as it can significantly affect physical and mechanical characteristics. PHA molecules with higher molecular weights are desirable for practical applications, especially considering that decreases in molecular weight have been reported during PHA extraction and purification processes [38], [39]. Moreover, PHAs with higher molecular weights are known to possess better mechanical properties [40]. The PHAs with molecular weight of more than 1× 10^6^ g/mol are categorized as ultra-high-molecular-weight PHA. Higher plants and *E*. *coli* lacking the *phaZ* gene have been shown to produce ultra-high-molecular-weight PHAs [41, 42]. Ultra-high-molecular-weight PHAs of approximately 2,000,000 g/mol can be produced by recombinant *E*. *coli* under specific conditions [41]. Sugarcane can produce PHB with molecular weights greater than 2,000,000 g/mol [42]. In the current study, we found that certain photosynthetic purple bacteria could produce PHAs with high molecular weights under natural nutrient-limited conditions. We believe these novel findings offer significant advantages for the production of PHAs for industrial applications.

**Fig 5:**
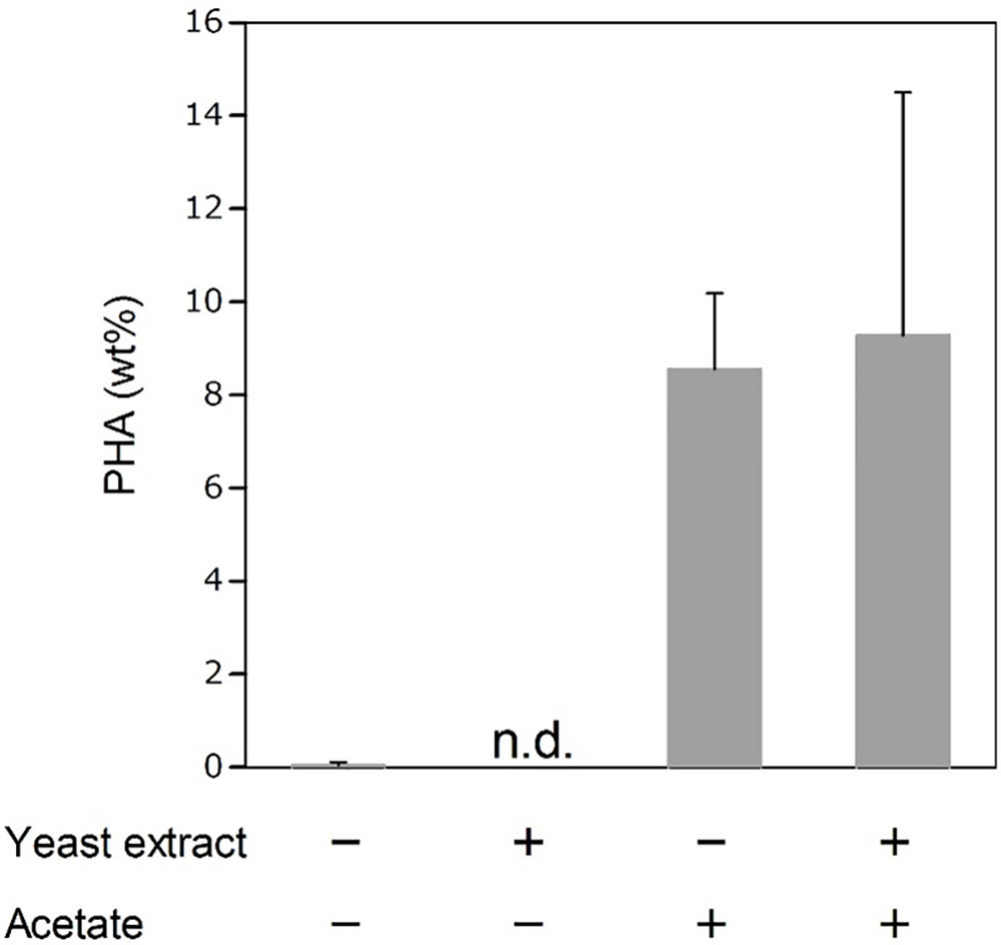
PHA content (wt%) of photosynthetic bacteria cultured in seawater. *Rdv*.*sulfidophilum* cells were cultured in growth medium until the cell cultures reached an approximate OD_660_ value of 1.0 and then diluted to an OD_660_ of 0.1 in s supplemented with 0.5% sodium acetate and/or 0.4 g/L of yeast extract. After 7 days, the cells were harvested, and intracellular PHA levels were characterized using GC-MS. Intracellular PHAs under growth conditions were analyzed using cells showing approximately OD_660_ = 1.0. n. d., not detected. Data are the mean ± SD of at least three cultures. *Values that show significant differences compared to the growth conditions (p<0.05).

The use of seawater as culture medium is one way to reduce the production cost of cultivation. PHA production was investigated using artificial seawater. The log phase cell cultures of *Rdv*. *sulfidophilum* were diluted to an OD_660_ of 0.1 in seawater with or without yeast extract and 0.5% acetate. The cells were harvested after 7 days, and intracellular PHA levels were characterized by GC-MS (Fig 5). CDW of cells cultured only in seawater was nearly identical to that of pre-cultured cells (S4 Fig). Seawater contained only low levels of nutrients especially carbon, nitrogen, phosphate and iron [43]. This suggests that photosynthetic purple bacteria could not grow well in seawater due to deficiency of nutrients. *Rdv*. *sulfidophilum* cells did not accumulate PHA only in seawater because of poor cell growth (Fig 5). CDWs were increased by the addition of yeast extract and acetate (S4 Fig). The addition of yeast extract did not lead to PHA production despite of recovery of the cell growth. On the other hand, *Rdv*. *sulfidophilum* cells accumulated 9 wt% PHA by the addition of acetate. The addition of both yeast extract and acetate did not enhance PHA production. These results indicate that acetate is a good carbon source for PHA production in seawater. The yield of PHA was 16 and 21 mg/L culture in seawater supplemented with acetate and both acetate and yeast extract, respectively. Thus, we demonstrated that PHA production could be achieved using seawater in photosynthetic purple bacteria. Optimization of culture conditions such as light conditions (light intensity and light quality) and carbon concentrations will be required to increase the CDW and PHA content using seawater. Two-stage cultivation system to separate PHA production from bacterial growth might also be effective for increasing the PHA content.

According to a previous study on life cycle assessment (LCA) of PHA [44], costs for the PHA production using a recombinant strain of *Ralstonia eutropha* and life cycle inventories of energy consumption and CO_2_ emissions have been calculated. Based on the LCA report, we evaluated the PHA production costs using photosynthetic purple bacteria. Photosynthetic purple bacteria can grow photoautotrophically, resulting the reducing the cost of carbon source because of utilization of photosynthetic product. In addition, well-known PHA-producing bacteria such as *R*. *eutropha* and *E*. *coli* are cultivated at 30–37°C, while photosynthetic purple bacteria are grown at around 25°C. Under this growth temperature, we spend less energy to keep the temperature of cell culture, leading to reducing the cost due to saving of electricity. Although lighting electricity is needed for growth of photosynthetic purple bacteria, LED is extremely efficient and solar light does not need any electricity. In the LCA for PHA production, cost for ammonia is set to 0.20 USD/kg, whereas other minerals are set to 0.004 USD/kg [44]. In the case of photosynthetic purple bacteria, nitrogen cost can be reduced because our study suggests that candidate strains have the nitrogen fixation ability. Furthermore, the use of seawater for PHA production by photosynthetic purple bacteria can reduce the cost of culture medium (Fig 5). On the basis of these factors, photosynthetic purple bacteria have several advantages over soil bacteria and are potentially applicable to industrial PHA production.

## Conclusion

In this study, we found that all photosynthetic purple bacteria tested were able to accumulate PHAs. PHA production had been previously reported in *Rdv*. *sulfidophilum* [13], whereas this is the first report of PHA production in the other 11 photosynthetic purple bacteria. Purple sulfur bacteria accumulated the PHB homopolymer. Some purple non-sulfur bacteria did not increase PHA content under nitrogen-limited conditions, implying that nitrogen was not limited in these strains, likely due to strong nitrogen fixation activity. All of the purple non-sulfur photosynthetic bacteria were able to produce copolymers consisting of 3HB and 3HV units. GPC analysis revealed that some of the photosynthetic purple bacteria synthesized high-molecular-weight PHA molecules due to decreased level of PhaC and PhaZ, which is an important property for industrial production. We also demonstrated that photosynthetic purple bacteria synthesized PHA in seawater supplemented only with acetate. We believe that the candidate photosynthetic purple bacteria strains identified in this study will be useful host microorganisms for industrial PHA production using marine resources.

## Materials and Methods

### Culture conditions

The photosynthetic purple bacteria investigated in this study were obtained from biological resource centers (RIKEN BioResource Center, DSMZ and ATCC), as listed in S1 Table. Purple sulfur bacteria were cultured in growth medium containing the following components per liter: KH_2_PO_4_ 0.5 g, CaCl_2_·2H_2_O 0.15 g, MgSO_4_·7H_2_O 2.0 g, NH_4_Cl 0.64 g, NaCl 20 g, sodium pyruvate 3.0 g, yeast extract 0.4 g, vitamin B12 2 mg, Na_2_S·9H_2_O 240 mg, Na_2_S_2_O_3_·5H_2_O 1.5 g, FeCl_2_·6H_2_O 1.5 mg, ZnCl_2_·5H_2_O 70 μg, MnCl_2_·4H_2_O 100 μg, H_3_BO_3_ 62 μg, CoCl_2_·6H_2_O 190 μg, CuCl_2_·2H_2_O 17 μg, NiCl_2_·6H_2_O 24 μg, and Na_2_MoO_4_·H_2_O 36 μg. The pH of the medium was adjusted to 7.5. Purple non-sulfur bacteria were cultured in growth medium containing the following components per liter: KH_2_PO_4_ 0.5 g, CaCl_2_·2H_2_O 0.25 g, MgSO_4_ ·7H_2_O 3.0 g, NH_4_Cl 0.68 g, NaCl 20 g, sodium malate 3.0 g, sodium pyruvate 3.0 g, yeast extract 0.4 g, ferric citrate 5 mg, vitamin B12 2 mg, ZnCl_2_·5H_2_O 70 μg, MnCl_2_·4H_2_O 100 μg, H_3_BO_3_ 60 μg, CoCl_2_·6H_2_O 200 μg, CuCl_2_·2H_2_O 20 μg, NiCl_2_·6H_2_O 20 μg, and Na_2_MoO_4_·H_2_O 40 μg. The pH was adjusted to 6.8. Photosynthetic purple bacteria were grown under continuous far-red LED light conditions (730 nm, 8 Wm^-2^) at 30°C in screw-capped bottles with continuous stirring using a multi-position stirrer.

For PHA production, NH_4_Cl, sodium malate and sodium pyruvate were removed from the growth medium, and 1 g sodium bicarbonate (NaHCO_3_) and/or 5 g sodium acetate per liter were added as carbon sources. For PHA production using seawater, Photosynthetic purple bacteria were cultured in 33.4 g/L of RED SEA SALTS (Red Sea U.S.A., Houston, USA) with or without the addition of 0.4 g/L yeast extract and 0.5% sodium acetate. Photosynthetic purple bacteria cells were cultured in growth medium and harvested during log phase (OD_660_ = ~1.0). The cells were harvested and washed with PHA production medium. The washed cells were diluted to a starting OD_660_ of 0.1 in PHA production medium and cultured for 7 days. After 7 days, the cells were harvested and washed with 20 mM Tris-HCl (pH 7) and then lyophilized.

### Analysis of PHA content and composition

The PHA content characterization method was modified slightly from a previous study [45]. Approximately 0.5–2 mg lyophilized cells were incubated in 100% ethanol at 70°C for 1 h to remove pigments. The cells were subjected to ethanolysis in the presence of 250 μL chloroform, 100 μL hydrochloric acid and 850 μL ethanol at 100°C for 4 h. After cooling, phosphate buffer (pH 8.1) was added to the reaction mixture and then neutralized with 0.65 N NaOH. After centrifugation at 1,500 rpm for 5 min, the lower chloroform layer was filtered through anhydrous sodium sulfate and incubated with molecular sieves for 30 min. PHA content and composition were determined using a gas chromatography–mass spectrometry (GC-MS) apparatus (GCMS-QP2010 Ultra, Shimadzu, Tokyo, Japan) equipped with a 30 m × 0.25 mm DB-1 capillary gas chromatography column (Agilent Technologies, CA, USA). For analysis, a 1-μL volume of sample solution was injected with helium as a carrier gas (3.30 mL min^-1^). The following temperature program was used to separate ethyl esters: 45°C for 1 min, temperature ramp of 7°C per min to 117°C. The interface and ion source temperatures were 250°C and 230°C, respectively. The 3HB content was determined using a calibration curve. The relative amount of 3-hydroxyvalerate (3HV) was estimated using a calibration curve of 3HB. The PHA content was calculated as a percentage of dry cell weight.

### Extraction of PHAs

PHAs were extracted from lyophilized cells using chloroform. The chloroform extracts were filtered and concentrated using a rotary vacuum evaporator. The chloroform-extracted PHAs were purified by precipitation with hexane. The PHA precipitate was filtered and then air-dried overnight. PHAs dissolved in chloroform were concentrated again using a rotary vacuum evaporator and purified by precipitation with cold methanol. The PHA precipitate was filtered and then air-dried overnight again. The purified PHAs were dissolved in chloroform and used for further analysis.

### Chemical structures and molecular weights of PHAs

The purified PHAs were analyzed by proton nuclear magnetic resonance (^1^H NMR) (JNM-Excalibur 270; JEOL, Ltd., Tokyo, Japan) to determine their chemical structures and compositions. The sample for NMR analysis was dissolved in CDCl_3_ with 0.05% (v/v) tetramethylsilane (TMS) (Wako Pure Chemical Industries Ltd., Osaka, Japan) at concentration of 4 mg/mL. The molecular weight of the PHAs was determined by gel-permeation chromatography (GPC) (RI-2031, PU-2086, AS-2055, CO-2056; JASCO, Tokyo, Japan) using Shodex K-806M, K802 and K-G columns at 40°C, according to a previously described method [46]. Chloroform was used as the mobile phase at a flow rate of 0.8 mL/min. The concentration of the purified PHAs was approximately 1 mg/mL. The molecular weight and polydispersity index (PDI) of the PHAs were estimated by comparison with polystyrene standards of the following molecular weights: 1.32 × 10^3^, 3.25 × 10^3^, 1.01 × 10^4^, 2.85 × 10^4^, 6.60 × 10^4^, 1.56 × 10^5^, 4.60 × 10^5^, 1.07 × 10^6^, and 3.15 × 10^6^ g/mol, respectively.

### Thermal properties of PHAs

Thermal properties of the PHAs were analyzed by differential scanning calorimetry (DSC). DSC measurements were performed by DSC8500 (PerkinElmer, Waltham, MA, USA). The PHAs were weighed in an aluminum pan and heated to 200°C at 20°C/min. The melting temperature (Tm) was determined from the DSC endotherms. The glass transition temperature (Tg) was calculated as the midpoint of the heat capacity change based on the DSC curves from the second heating.

### Quantitative reverse transcription polymerase chain reaction (RT-PCR) analysis

RNA of *Rdv*. *sulfidophilum* was extracted under growth and nitrogen-limited conditions. Cells were cultured in growth medium until the cell cultures reached an approximate OD_660_ value of 1.0 and RNA under growth conditions was extracted. These cultures were diluted to an OD_660_ of 0.1 in nitrogen-limited media supplemented with 0.5% sodium acetate. After 7 days, the cells were harvested, and RNA under nitrogen-limited conditions was extracted. Total RNA was extracted from *Rdv*. *sulfidophilum* cells using RNAeasy Mini kit (QIAGEN, Tokyo, Japan). Using the 0.1 μg RNA as a template, cDNA was synthesized by QuantiTect Reverse Transcription Kit (QIAGEN, Tokyo, Japan) following the manufacturer's protocol. The PCR was performed using SsoAdvanced^™^ Universal SYBR Green Supermix (BIO-RAD, Hercules, USA) and the product was analyzed by StepOne (ThermoFisher SCIENTIFIC, Yokohama, Japan) according to the protocol. To determine expression levels of the *phaC* gene, *phaP* gene, *phaZ* gene and *rpoD* gene, the following sets of primers were used: *phaC*, phaC-F (5’- ATTGAGCCCGTCGATATCCT -3’) and phaC-R (5’- GCA GAC CCA TCC CTA TTT CA -3’); *phaP*, phaP-F (5’- TCG ACG ATC TTA ACG TCC CT -3’) and phaP-R (5’- CAA TAC GGA GAC CAG CGA TT -3’); *phaZ*, phaZ-F (5’- TGC GAC GTC TAT ATC ACC GA -3’) and phaZ-R (5’- CCG AGA TGC TTG AGG AAA TC -3’); *rpoD*, rpoD-F (5’- CTT GTC CTC GAT GAA ATC GC -3’) and rpoD-R (5’- GTC CGC AAG GTG ATG AAG AT -3’).

### Transmission electron microscopy

Cells from log phase cultures grown in growth medium and in nitrogen-limited medium containing sodium acetate were collected. The cells were fixed for transmission electron microscopy as described by Akai et al. [47]. Ultra-thin sections were examined using a transmission electron microscope (TEM) (JEM-1400; JEOL Ltd.) at 80 kV.

## Supporting Information

S1 Fig Cell dry weight of photosynthetic purple bacteria. Cell dry weights were measured when they were grown in growth medium (white bars), nitrogen limited medium containing both NaHCO_3_ and sodium acetate (light gray bars), nitrogen limited medium containing sodium acetate (light gray bars) and nitrogen limited medium containing NaHCO_3_ (black bars). Data are the mean ± SD of at least three cultures. *Values which show significant difference compared to the growth conditions (p<0.05).
(TIF)

S2 Fig PHA contents of photosynthetic purple bacteria. PHA contents were measured when photosynthetic purple bacteria were cultured in growth medium (white bars), nitrogen-limited medium containing both NaHCO_3_ and sodium acetate (light gray bars), nitrogen-limited medium containing only sodium acetate (dark gray bars) and nitrogen-limited medium containing only NaHCO_3_ (black bars). *Values with significant differences compared with the growth conditions (*p*<0.05). Data are the mean ± SD from at least three cultures.
(TIF)

S3 Fig GPC retention times of purified PHAs from *Mch*. *bheemlicum*, *Rdv*. *visakhapatnamense*, *Rdv*. *imhoffii* and *Rdv*. *euryhalinum*. Polystyrene standards (molecular weights: 3 148,843, 1 074 876, 460 595, 156 528, 66 001, 28 517, 10 112, 3252, and 1319 g/mol) was used for calibration.
(TIF)

S4 Fig Cell dry weights of photosynthetic bacteria cultured in seawater. Cell dry weights were measured when photosynthetic purple bacteria were cultured in seawater with or without 0.4 g/L of yeast extract and 0.5% sodium acetate. Data are the mean ± SD from at least three cultures.
(TIF)

S1 Table Photosynthetic purple bacteria strains used in this study. (PDF)

S2 Table Thermal properties of the purified PHAs. (PDF)
