# Supplementary figures and images for: Synthesis of High-Molecular-Weight Polyhydroxyalkanoates by Marine Photosynthetic Purple Bacteria
Source: PLoS One. 2016 Aug 11;11(8):e0160981. doi: 10.1371/journal.pone.0160981 (PMC4981452; doi:10.1371/journal.pone.0160981)

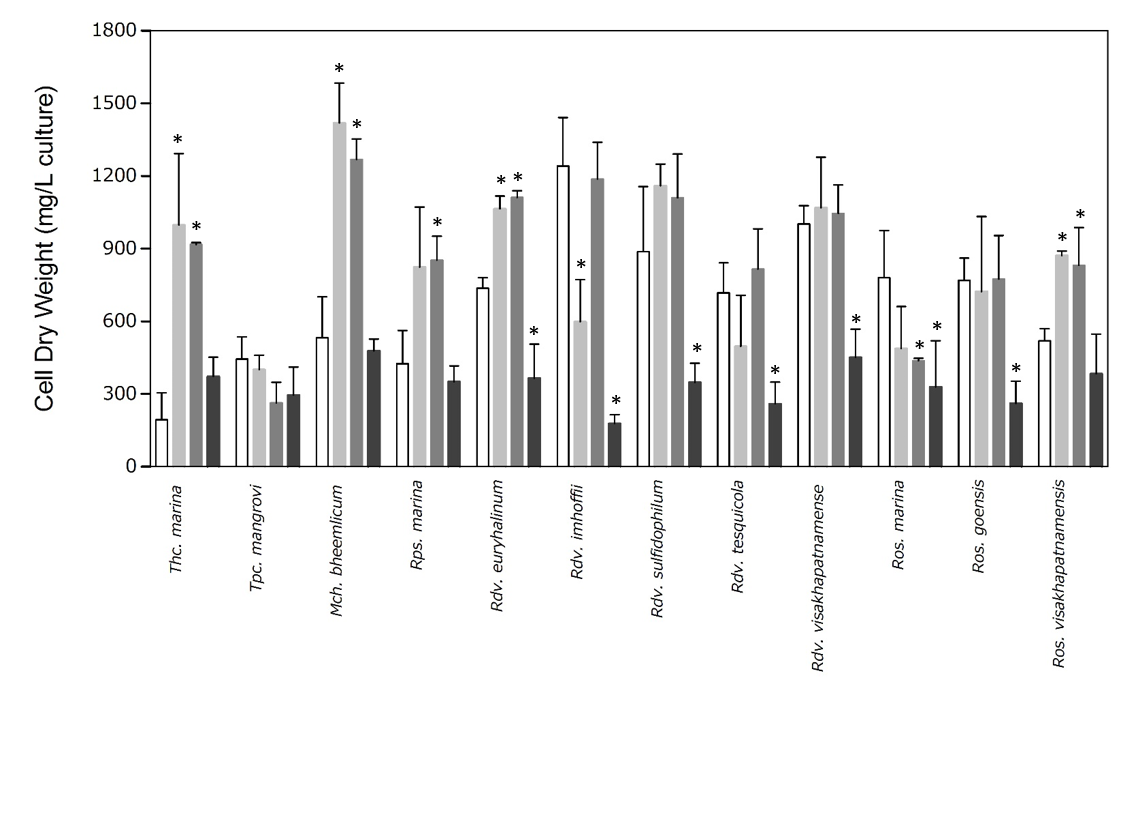

Supplement: S1 Fig — Cell dry weights were measured when they were grown in growth medium (white bars), nitrogen limited medium containing both NaHCO3 and sodium acetate (light gray bars), nitrogen limited medium containing sodium acetate (light gray bars) and nitrogen limited medium containing NaHCO3 (black bars). Data are the mean ± SD of at least three cultures. *Values which show significant difference compared to the growth conditions (p<0.05). (TIF) [file pone.0160981.s001.tif]

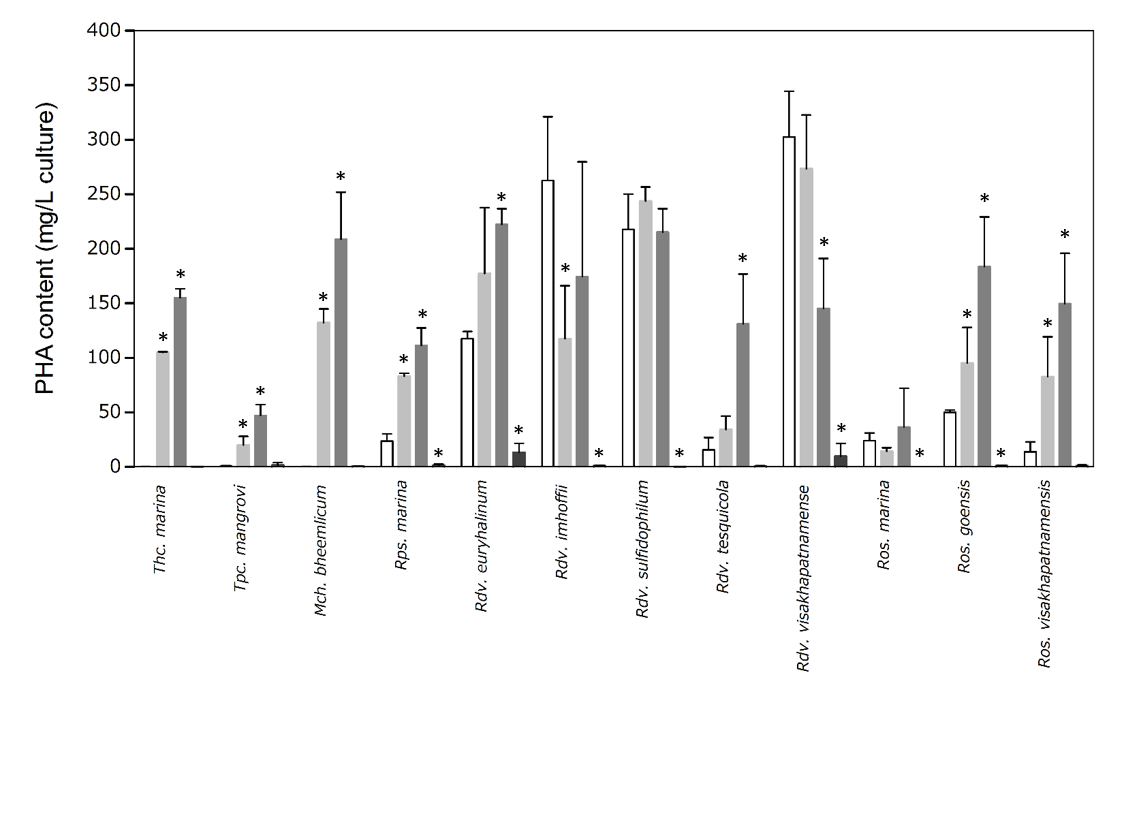

Supplement: S2 Fig — PHA contents were measured when photosynthetic purple bacteria were cultured in growth medium (white bars), nitrogen-limited medium containing both NaHCO3 and sodium acetate (light gray bars), nitrogen-limited medium containing only sodium acetate (dark gray bars) and nitrogen-limited medium containing only NaHCO3 (black bars). *Values with significant differences compared with the growth conditions (p<0.05). Data are the mean ± SD from at least three cultures. (TIF) [file pone.0160981.s002.tif]

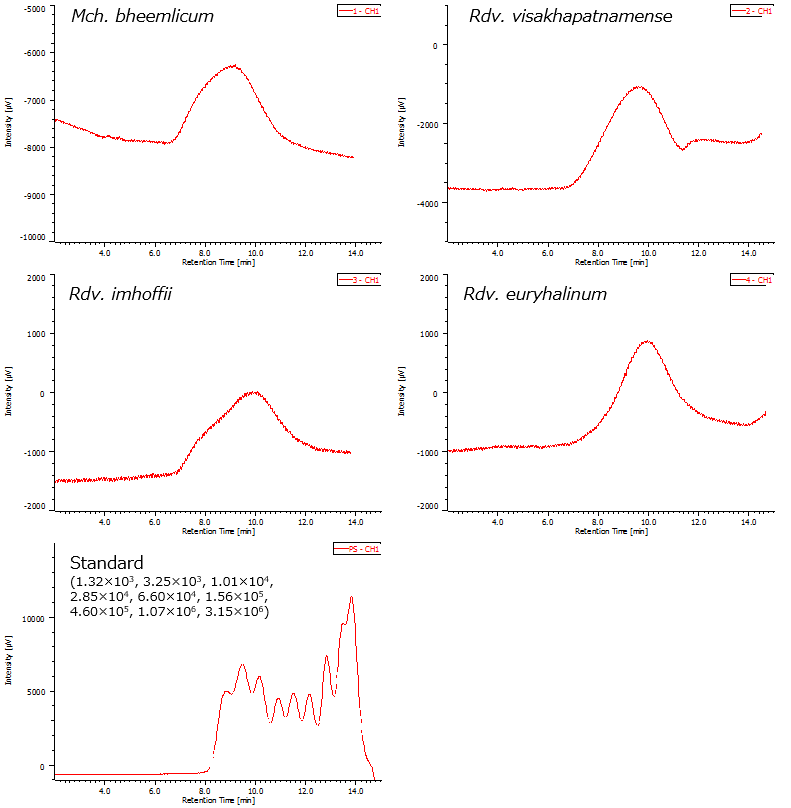

Supplement: S3 Fig — Polystyrene standards (molecular weights: 3 148,843, 1 074 876, 460 595, 156 528, 66 001, 28 517, 10 112, 3252, and 1319 g/mol) was used for calibration. (TIF) [file pone.0160981.s003.tif]

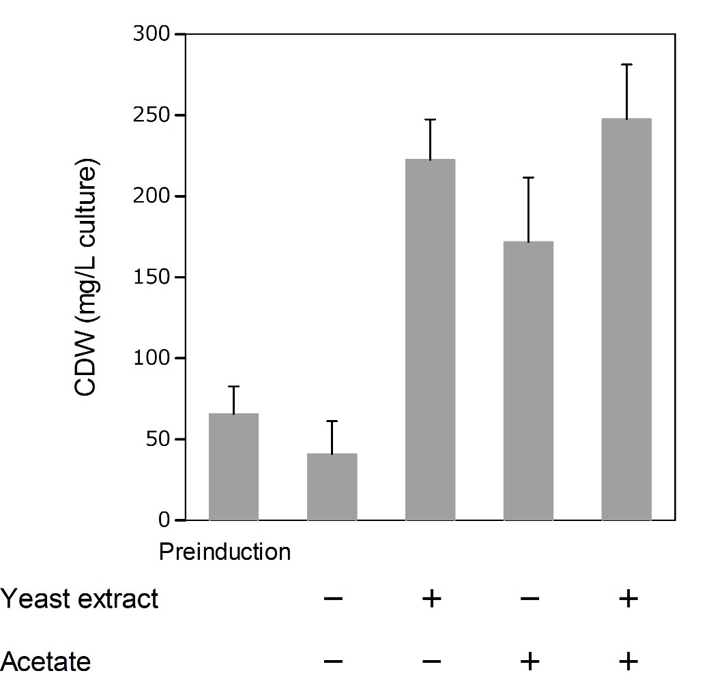

Supplement: S4 Fig — Cell dry weights were measured when photosynthetic purple bacteria were cultured in seawater with or without 0.4 g/L of yeast extract and 0.5% sodium acetate. Data are the mean ± SD from at least three cultures. (TIF) [file pone.0160981.s004.tif]
