# Supplementary material for: Synthesis of High-Molecular-Weight Polyhydroxyalkanoates by Marine Photosynthetic Purple Bacteria
Source: PLoS One. 2016 Aug 11;11(8):e0160981. doi: 10.1371/journal.pone.0160981 (PMC4981452; doi:10.1371/journal.pone.0160981)
Supplement: S1 Table — (PDF) [file pone.0160981.s005.pdf]

S1 Table. Photosynthetic purple bacteria strains used in this study.

Resource number	Scientific name	Growth temperature
JCM14796	*Allochromatium phaeobacterium*	30℃
JCM14262	*Allochromatium renukae*	28℃
ATCC51935	*Ectothiorhodospira haloalkaliphila*	30℃
DSM241	*Ectothiorhodospira marina*	25℃
DSM237	*Ectothiorhodospira mobilis*	35℃
BRC14151	*Halochromatium roseum*	30℃
DSM244	*Halorhodospira halophila*	25℃
JCM17835	*Halorhodospira neutriphila*	30℃
JCM13911	*Marichromatium bheemlicum*	28℃
DSM1591	*Marichromatium purpuratum*	25℃
JCM13533	*Marichromatium sp.*	26℃
ATCC700894	*Thiocapsa litoralis*	26℃
JCM14780	*Thiohalocapsa marina*	25℃
JCM14889	*Thiophaeococcus mangrovi*	30℃
JCM14149	*Thiorhodococcus bheemlicus*	26℃
JCM14150	*Thiorhodococcus kakinadensis*	28℃
DSM19920	*Rhodobaca barguzinensis*	28℃
JCM14544	*Rhodobacter vinaykumarii*	30℃
JCM13532	*Rhodobium gokarnense*	26℃
JCM9337	*Rhodobium orientis*	26℃
ATCC BAA1145	*Afifella pfennigii (Rhodobium pfennigii)*	30℃
DSM2698	*Afifella marina (Rhodobium marinum)*	25℃
DSM2132	*Rhodothalassium salexigens*	26℃
DSM2781	*Rhodovulum adriaticum*	26℃
DSM4868	*Rhodovulum euryhalinum*	30℃
JCM13300	*Rhodovulum marinum*	30℃

JCM13589	*Rhodovulum imhoffii*	28℃
ATCC35886	*Rhodovulum sulfidophilum*	28℃
ATCC BAA1573	*Rhodovulum tesquicola*	30℃
JCM13531	*Rhodovulum visakhapatnamense*	30℃
BRC14191	*Roseospira goensis*	28℃
ATCC BAA447	*Roseospira marina*	26℃
ATCC BAA1365	*Roseospira visakhapatnamensis*	26℃
