# Supplementary material for: Synthesis of High-Molecular-Weight Polyhydroxyalkanoates by Marine Photosynthetic Purple Bacteria
Source: PLoS One. 2016 Aug 11;11(8):e0160981. doi: 10.1371/journal.pone.0160981 (PMC4981452; doi:10.1371/journal.pone.0160981)
Supplement: S2 Table — (PDF) [file pone.0160981.s006.pdf]

S2 Table. Thermal properties of the purified PHAs.

Organism	T_g (°C)	T_m (°C)	Number-average molecular weight (g/mol)	3HB composition (mol%)	3HV composition (mol%)
Thc. marina	n.d.	172	645×10^3	100	0
Tpc. mangrovi	-3	171	72×10^3	100	0
Mch. bheemlicum	-1	174	994×10^3	100	0
Rps. marina	n.d.	142	4×10^3	93.9 ± 5.3	6.1 ± 5.3
Rdv. euryhalinum	-1	168	447×10^3	97.2 ± 0.9	2.8 ± 0.9
Rdv. imhoffii	-2	165	570×10^3	96.8 ± 3.2	3.2 ± 3.2
Rdv. sulfidophilum	-2	162	300×10^3	92.6 ± 1.6	7.4 ± 1.6
Rdv. tesquicola	n.d.	165	504×10^3	64.9 ± 16.5	35.1 ± 16.5
Rdv. visakhapatnamense	n.d.	n.d.	713×10^3	98.4 ± 1.5	1.6 ± 1.5
Ros. marina	n.d.	132	3×10^3	70.6 ± 7.0	29.4 ± 7.0
Ros. goensis	n.d.	74	17×10^3	34.0 ± 8.9	66.0 ± 8.9
Ros. visakhapatnamensis	-4	159	147×10^3	69.9 ± 11.1	30.1 ± 11.1

n.d., not detected.
